# Untargeted Plasma Metabolomics Reveals Metabolic Differences Associated with Job Stress in Manufacturing Workers: A Cross-Sectional Study

**DOI:** 10.3390/metabo16100790

**Published:** 2026-10-18

**Authors:** Guofeng Li, Jing Liao, Peifang Liu, Yifei Liu, Yulan Chen, Chuancheng Wu, Jing Li, Bo Shen, Yu Jiang

**Affiliations:** 1 Department of Preventive Medicine, School of Public Health, Fujian Medical University, Fuzhou 350108, China; 2 The Key Laboratory of Environment and Health, School of Public Health, Fujian Medical University, Fuzhou 350108, China; 3 Fuzhou Municipal Center for Disease Control and Prevention, Fuzhou 350200, China

**Keywords:** job stress, untargeted metabolomics, plasma, lipid metabolism, LC–MS, cross-sectional study

## Abstract

**Background**: Job stress is a common occupational hazard and has been linked to adverse mental and cardiometabolic outcomes; however, the metabolic responses to job stress remain incompletely understood. This study aimed to characterize plasma metabolic alterations associated with job stress and identify the potential metabolic pathways involved. **Methods**: A total of 195 male manufacturing workers aged 20–60 years were recruited in Fuzhou, China. After applying the inclusion and exclusion criteria and screening for outliers, 143 participants were included in the final analysis, comprising 78 workers in the job stress group and 65 in the non-job stress group. Job stress was assessed using the revised Chinese Job Content Questionnaire on the basis of the demand–control model, with a demand-to-control ratio > 1.0 warranting inclusion in the stress group. Fasting plasma samples were analyzed through untargeted liquid chromatography–mass spectrometry (LC–MS). Differentially abundant metabolites were identified using univariate and multivariate statistical approaches, and pathway enrichment analyses were conducted using MetaboAnalyst 6.0. **Results**: Twelve plasma metabolite features, predominantly lipid species such as phosphatidylethanolamine (PE), diacylglycerol (DG), and free fatty acids, were significantly associated with job stress after adjustment for covariates. Five features (M573T1, M264T3, M673T5, M686T27 and M569T1) showed moderate discriminative ability (AUC > 0.7) for job stress exposure. Pathway analysis revealed enrichment of seven metabolic pathways, among which glycerophospholipid metabolism was significantly enriched (FDR_*P* < 0.05). **Conclusions**: Untargeted plasma metabolomics revealed distinct metabolic differences associated with job stress, particularly in relation to lipid metabolism and membrane remodeling pathways. These findings provide preliminary evidence supporting the utility of metabolomics in exploring occupational stress-related metabolic signatures and identifying exploratory candidate markers for further validation.

## 1. Introduction

Job stress is a psychological and physiological response that occurs when the demands of work exceed an individual’s perceived resources, autonomy, or coping capacity [1]. In modern occupational settings, job stress has become increasingly prevalent and is now recognized as an important contributor to both mental and physical health burdens. A study investigating job stress among hospital residents revealed that out of 278 respondents, 169 (60.8%) had moderate stress levels and 105 (37.8%) had high stress levels [2]. Another epidemiological investigation revealed that among 9658 participants, 26.7% were exposed to low levels of job stress and 18.1% to high levels of job stress [3]. Job stress is associated with many adverse physical and mental health outcomes [4], such as depression [5,6], cardiovascular disease [7], type 2 diabetes mellitus (T2DM) [8] and cancer [9,10]. Because the adverse consequences of job stress often develop gradually and involve multiple organ systems, there is increasing interest in identifying early biological changes that may precede overt disease.

Metabolomics offers a strategy for capturing the downstream biochemical consequences of exposure to environmental and psychosocial factors [11,12]. Unlike approaches quantifying a single biomarker, metabolomic profiling can provide a systems-level view of metabolic adaptation and dysregulation, helping to reveal pathways through which stress influences disease susceptibility [13]. In particular, plasma metabolites are dynamic indicators of host physiology and can reflect alterations in energy metabolism, lipid remodeling, oxidative balance, and inflammatory signaling [14,15,16]. Previous metabolomics studies have shown that stress-related conditions are often accompanied by altered lipid and amino acid metabolism, suggesting that stress exposure may be accompanied by measurable metabolic changes [17].

Despite these advances, the metabolic alterations associated with job stress remain insufficiently characterized, especially among occupational groups with substantial daily work demands. Most existing studies have focused on questionnaire-based assessments of psychological symptoms or on traditional risk factors such as blood pressure and dyslipidemia [18,19,20]. These approaches, while valuable, do not fully explain the biological pathways linking psychosocial stress to long-term disease risk. In addition, the metabolic differences associated with job stress may differ by occupation, sex, lifestyle, and exposure intensity, further underscoring the need for population-specific investigations.

Manufacturing workers represent an important population for occupational stress research, as they are frequently exposed to repetitive production demands, shift work requirements, time constraints, and other work-related psychosocial burdens. Given this context, in this study, untargeted plasma metabolomics analysis was performed to explore metabolic features associated with occupational stress in manufacturing workers, evaluate the utility of these features as candidate discriminatory features for occupational stress, and delineate the primary metabolic pathways linked to stress exposure. The aim of this study is to deepen the understanding of biochemical alterations induced by occupational stress through the integration of liquid chromatography–mass spectrometry (LC–MS)-based metabolic profiling with pathway enrichment analysis, laying a theoretical foundation for future mechanistic and preventive investigations.

## 2. Materials and Methods

### 2.1. Study Population

This cross-sectional study was approved by the Ethics Committee of Fujian Medical University (No. [2019]025) and was conducted in accordance with the Declaration of Helsinki. Written informed consent was obtained from all participants before enrollment.

The study was carried out from June to December 2020 in Fuzhou, Fujian Province, China. A total of 195 male manufacturing workers aged 20–60 years were initially recruited. The inclusion criteria for participants were as follows: (1) aged 20 to 60 years; (2) work tenure of at least 1 year; and (3) completion of a face-to-face questionnaire covering demographic characteristics and job stress-related information and provision of blood samples. The exclusion criteria were as follows: (1) self-reported or physician-diagnosed metabolic disease and (2) use of medications that may affect metabolism, including dexamethasone, estrogen, and glucocorticoids, in the previous 3 months. Eleven participants were excluded for having a work tenure of less than 1 year, 7 for having a previous diagnosis of metabolic disease, 9 for having taken medications that may affect metabolism (including dexamethasone, estrogen, and glucocorticoids) within the past 3 months, and 10 for lacking questionnaire or blood data (only one type of data was provided). Ultimately, 37 participants were excluded according to these criteria, and an additional 15 participants were excluded as outliers, resulting in a final sample of 143 workers for analysis.

### 2.2. Assessment of Job Stress

Job stress was evaluated using the revised Chinese version of the Job Content Questionnaire (CJCQ), which has been shown to have good reliability and validity in Chinese populations [21,22]. The questionnaire includes 22 items covering three dimensions: job demand (5 items), job control (9 items), and social support (8 items). Items were rated on a 4-point Likert scale (1 = almost never, 2 = occasionally, 3 = often, 4 = always), and the score for each dimension was obtained by summing the scores of its constituent items [23]. In accordance with the demand–control model, the job stress ratio was calculated as the weighted job demand score divided by the weighted job control score.

$$D/C=\frac{\mathrm{job}\;\mathrm{demand}\;\mathrm{score}}{\mathrm{job}\;\mathrm{control}\;\mathrm{score}\times A}$$

A represents the ratio of the number of work demand items to the number of work control items, which is 5/9.

Participants with a ratio greater than 1.0 were included in the job stress group, whereas those with a ratio ≤ 1.0 were included in the non-job stress group [24]. The Cronbach’s α coefficient for the reliability analysis of the 22 items of the CJCQ was 0.928.

### 2.3. Blood Sample Collection and Plasma Preparation

After the participants fasted for 8 h, venous blood samples were collected from each participant during the occupational health examination. The samples were subsequently centrifuged at 3000 rpm for 10 min and stored at −80 °C until analysis.

For plasma preparation, 150 μL of plasma was transferred to a 1.5 mL Eppendorf tube (Haige Biotechnology Co., Ltd., Fuzhou, China), followed by the addition of 300 μL of acetonitrile (chromatography grade, Merck, Germany). The mixture was vortexed for 30 s and allowed to stand at 4 °C for 10 min. A second 150 μL aliquot of acetonitrile was then added, followed by additional vortexing and incubation at 4 °C for 3 h to precipitate the plasma proteins completely. The samples were subsequently centrifuged at 1600 rpm at 4 °C for 30 min, after which the upper layer of the supernatant was transferred to a new tube. After filtration through a 0.22 μm membrane (Haige Biotechnology Co., Ltd., Fuzhou, China), 100–150 μL of the clarified solution was transferred to LC–MS vials for analysis.

Quality control (QC) samples were prepared by pooling equal aliquots from all plasma samples. QC samples were analyzed at the beginning of the run, during the analytical sequence, and at the end of the run to monitor instrument stability and reproducibility.

### 2.4. LC–MS Analysis

Plasma metabolite profiling was performed using an Agilent 1200 HPLC system coupled to an Agilent 6520 accurate-mass quadrupole time-of-flight mass spectrometer equipped with a dual electrospray ionization (Dual ESI) source (Agilent Technologies, Santa Clara, CA, USA). Chromatographic separation was carried out on an Agilent SB-C18 column (2.1 × 50 mm, 1.8 μm; Agilent Technologies, Santa Clara, CA, USA) maintained at 37 °C, with an injection volume of 20 μL. Mobile phase A consisted of ultrapure water containing 0.1% (*v*/*v*) formic acid, and mobile phase B consisted of acetonitrile containing 0.1% (*v*/*v*) formic acid (Merck, Germany). The flow rate was set at 0.25 mL/min. Prior to use, the mobile phases were degassed by ultrasonication in a KQ-250E ultrasonic cleaner (Kunshan, China). Separation was performed using the gradient program shown in Table 1. The column was equilibrated with the initial mobile phase for 30 min before each batch, and a column wash step was performed after each sample was analyzed as shown in Table 2.

Although a 20 μL injection volume is relatively large for this C18 column and may have affected the peak shape for some early-eluting compounds, this parameter was selected to maximize the sensitivity of untargeted plasma metabolomics analysis. The repeatability of chromatographic analysis and data reliability were ensured to a certain extent by rigorous QC procedures and system suitability testing.

The mass spectrometer was operated in full-scan mode over an m/z range of 50–1500. The Dual ESI source was used in both positive and negative ionization modes, with a capillary voltage (VCap) of 3000 V in both modes and a fragmentor voltage of 70 V. Nitrogen (Xinzhongming Chemical Co., Ltd., Fuzhou, China) was used as the drying and nebulizing gas at a drying gas temperature of 350 °C and a flow rate of 8.0 L/min, with a nebulizer pressure of 35 psig. The optimized source parameters are summarized in Table 3. Raw data were acquired using Agilent MassHunter Acquisition software (version B.07.00).

### 2.5. Quality Control and Data Preprocessing

To minimize potential batch effects, samples were injected in a randomized order, and rapid acquisition was performed. The entire dataset was acquired across 3 analytical batches over 10 days. To ensure analytical reliability, all the experimental procedures were conducted in accordance with standardized protocols. During metabolite profiling, one QC sample was injected after every 10 study samples, and 16 QC samples (accounting for 9.2% of all injections) were used as references for batchwise normalization. Blank samples were included to eliminate background signals; ions with intensities exceeding 5000 in positive-ion mode or 2000 in negative-ion mode in blank samples were regarded as background noise and excluded from further analysis. Initial filtering was then performed to address missing values, and 2370 features were retained. QC filtering based on the relative standard deviations (RSDs) of peak intensities in the QC samples was subsequently performed, and 60% had an RSD ≤ 20% (1422 out of 2370 retained features). When stratified by ionization mode, the RSD pass rate was 57% in positive-ion mode and 63% in negative-ion mode, indicating that the results were acceptable. Following this, 957 features were retained after manual EIC review (Figure S1).

Raw LC–MS data were converted to mzXML format using Agilent MassHunter Qualitative Analysis software (version B.07.02) and subsequently processed using XCMS Online (http://xcmsonline.scripps.edu) for peak detection, retention-time correction, peak alignment, and feature matching. Features with an RSD greater than 20% in the QC samples were removed. Features missing in more than 50% of the samples were excluded, and the remaining missing values were imputed with half of the minimum positive value. In terms of outliers, on the basis of OPLS-DA Hotelling’s T^2^ and DModX, samples exceeding the 99% T^2^Crit threshold and those exceeding twice the DCrit 0.05 threshold were excluded. The resulting data were then median-normalized, log-transformed, and subjected to downstream statistical analyses.

### 2.6. Metabolite Identification and Pathway Analysis

Metabolite annotation was performed by matching accurate mass (±10 ppm) against the HMDB (www.hmdb.ca) and KEGG (www.genome.jp/kegg/) databases. Pathway enrichment analysis was conducted using MetaboAnalyst 6.0; the 12 putatively annotated metabolites were compared against the KEGG pathway database to identify potential biological processes associated with occupational stress. The significance of enrichment was evaluated by a hypergeometric test, with nominal significance set at a raw *p* < 0.05.

### 2.7. Statistical Analysis

Statistical analyses were performed using R software (version 4.6.1) and MetaboAnalyst 6.0. Continuous variables are expressed as the mean ± standard deviation (mean ± SD), and group differences were assessed using Student’s *t* test. Categorical variables are expressed as frequencies, and group differences were assessed using the chi-square test. For metabolomics data, differentially abundant metabolites were identified using univariate analysis combined with multivariate statistics, and *p* values were adjusted for multiple comparisons using the false discovery rate (FDR) procedure. An FDR_q < 0.05 was considered to indicate statistical significance in the metabolomics analyses. To control for potential confounding factors, partial correlation analyses were conducted with adjustment for body mass index (BMI), education level, occupational tenure, shift status, smoking behavior, alcohol consumption, and exercise. Categorical covariates were incorporated into the model as dummy variables using *n*-1 coding, and the group with the lowest risk was set as the reference category for each variable to ensure the interpretability of effect size estimates. Receiver operating characteristic (ROC) curves were used to evaluate the discriminatory performance of exploratory candidate markers. For all other analyses, a two-tailed *p* value < 0.05 was considered to indicate statistical significance.

## 3. Results

### 3.1. Characteristics of the Study Participants

A total of 143 workers were included in the final analysis, comprising 78 participants in the job stress group and 65 in the non-job stress group. As shown in the original demographic comparisons, the two groups were generally comparable in terms of BMI, height, weight, education level, shift status, smoking behavior, alcohol consumption, and exercise habits (*p* > 0.05). However, occupational tenure differed significantly between the two groups, indicating that stress exposure may be related to occupational tenure (*p* < 0.05) (Table 4).

These findings suggest that the observed metabolic differences were unlikely to be explained solely by basic demographic imbalance and may represent physiological differences associated with job stress.

### 3.2. Plasma Metabolomic Profiling

Detection of peaks in the LC–MS data yielded 17,842 features, which were then subjected to alignment, matching, and missing-value filtering. In an ideal extracted ion chromatogram (EIC), the peak cluster should be relatively concentrated, meaning that the variation in ion intensity across samples should be confined to a narrow retention-time window, as shown in Figure S2A. However, some EICs exhibited a more dispersed peak cluster or multiple peak clusters, indicating retention-time shifts in ion intensity across samples, which in turn introduced bias into the calculation and annotation of the corresponding feature ion responses, as shown in Figure S2B. Therefore, unqualified EICs were screened and excluded one by one, and 709 feature ions in positive-ion mode and 248 feature ions in negative-ion mode were ultimately retained.

In addition, OPLS-DA was used to identify differences between the job stress group and the non-job stress group. As shown in Figure S3A, the score plot in positive-ion mode indicated clear separation between the two groups, with model parameters R^2^X(cum) = 0.498, R^2^Y(cum) = 0.924, and Q^2^(cum) = 0.721, all of which indicated the high reliability of the model predictions. In contrast, the corresponding parameters for the negative-ion mode score plot (Figure S3B) were R^2^X(cum) = 0.844, R^2^Y(cum) = 0.106, and Q^2^(cum) = 0.0138, suggesting no effective predictive discrimination between groups in this mode. Therefore, differentially abundant metabolites were subsequently screened solely on the basis of the data obtained in positive-ion mode. In positive-ion mode, the R^2^ intercept and Q^2^ intercept of the 200-permutation test were 0.582 and −0.612, respectively, indicating that the OPLS-DA model was valid and effective (Figure S3C).

### 3.3. Differential Metabolite Features Associated with Job Stress

Using univariate and multivariate statistical analyses, 23 differential features associated with occupational stress were identified using the thresholds of VIP > 1.0 and FDR_q < 0.05 (Table S1). On the basis of the accurate mass (±10 ppm), the m/z of these 23 features was matched and annotated against the HMDB, and exogenous nonhuman plasma substances, including plant-derived compounds, marine natural products, mycotoxins, drugs, and food pigments, were excluded, ultimately yielding 12 differentially annotated features putatively present in human plasma (Table S2). Given the absence of MS/MS data and validation using authentic standards, metabolite annotation was at MSI level 3. After adjusting for confounding factors, including BMI, education level, occupational tenure, shift status, smoking consumption, alcohol consumption, and exercise, all 12 features remained significantly associated (FDR_q < 0.05). Among these, M687T1 was positively correlated with occupational stress, whereas the other 11 characteristics were negatively correlated with occupational stress (Table 5).

### 3.4. ROC Analysis of Differential Features

**Figure 1:**
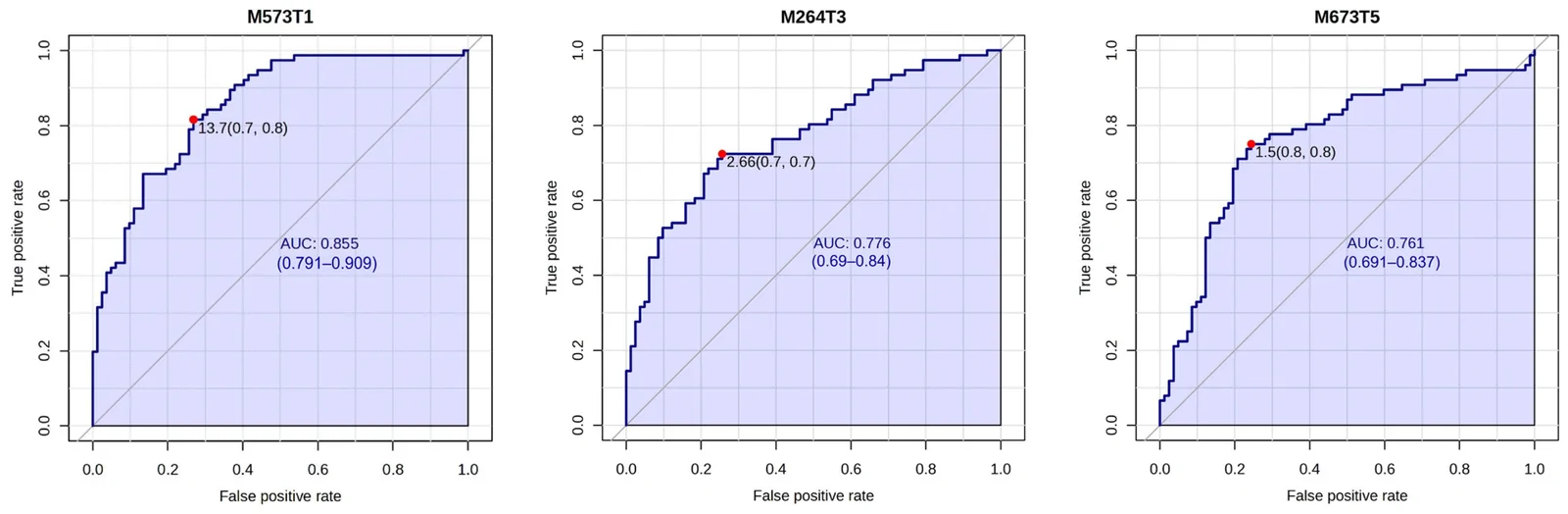

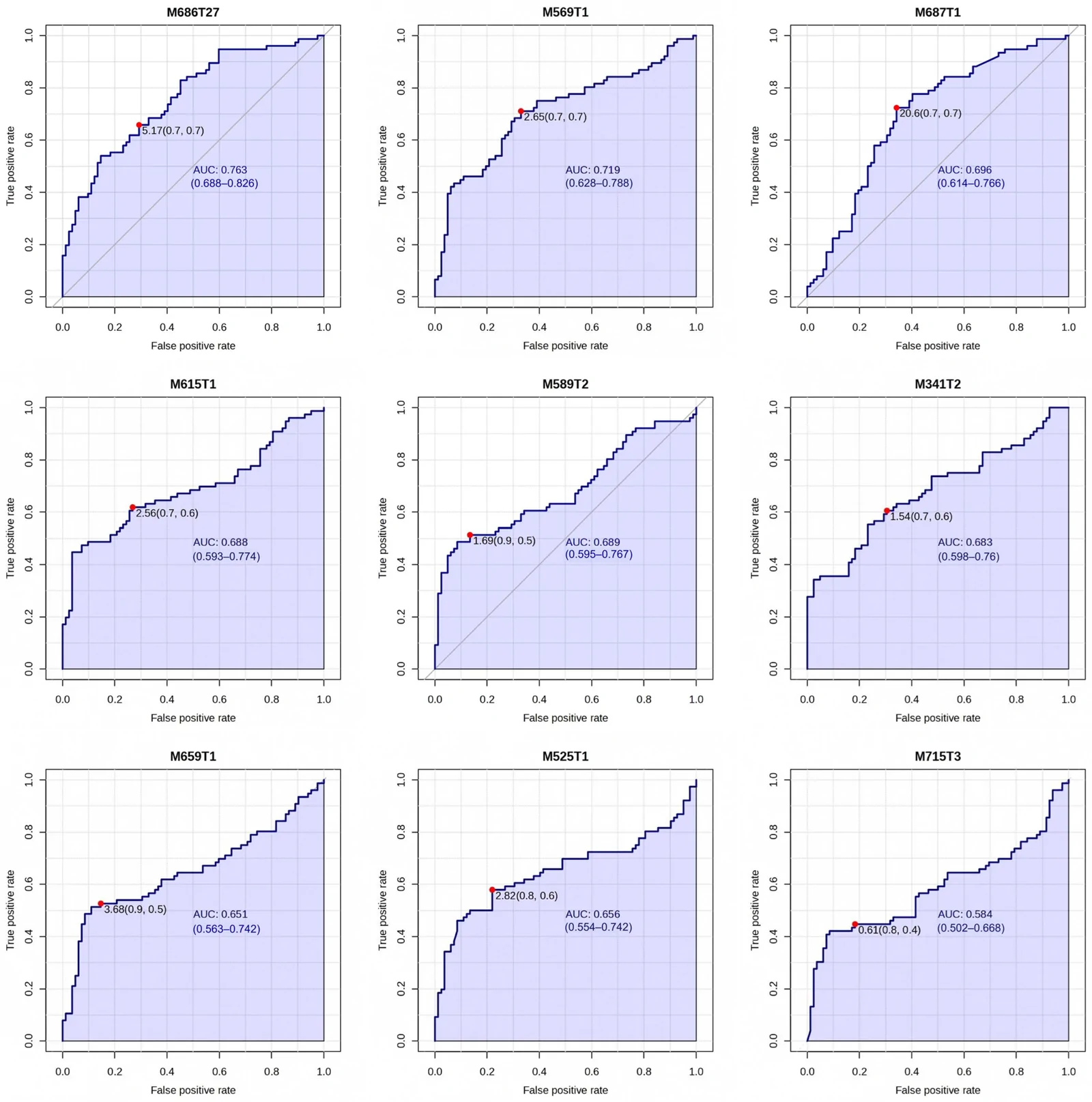
ROC curves of the 12 differential features associated with occupational stress (apparent AUC).

To evaluate the potential utility of differential features as candidate discriminatory features for occupational stress, we performed ROC curve analysis of 12 preliminarily annotated features using MetaboAnalyst 6.0, with the area under the curve (AUC) serving as the quantitative metric for discriminatory capacity (Figure 1). As shown in Table S3, the AUC values for all the features were significantly greater than 0.5 (*p* < 0.05). Notably, five features—M573T1, M264T3, M673T5, M686T27, and M569T1—exhibited AUC values exceeding 0.7, indicating moderate discriminatory power for occupational stress exposure. Furthermore, we conducted internal validation of the ROC curve analysis using MetaboAnalyst. Monte Carlo cross-validation (MCCV) revealed that the model comprising all 12 features had a CV-AUC of 0.94 (95% CI: 0.877–0.982), whereas the optimal 10-feature model (M264T3, M615T1, M341T2, M687T1, M573T1, M589T2, M673T5, M686T27, M569T1, M715T3) had a CV-AUC of 0.942 (95% CI: 0.89–0.986), with a prediction accuracy of 85.6%. The apparent AUC of this feature model was 0.938 (95% CI: 0.888–0.977), and 1000 permutations resulted in an empirical *p* value < 0.001, confirming that its discriminatory performance was significantly superior to chance. These findings suggest that the corresponding metabolites may serve as candidate discriminatory features for occupational stress, albeit further validation in an independent cohort with confirmation by MS/MS is warranted.

### 3.5. Pathway Enrichment Analysis

**Figure 2:**
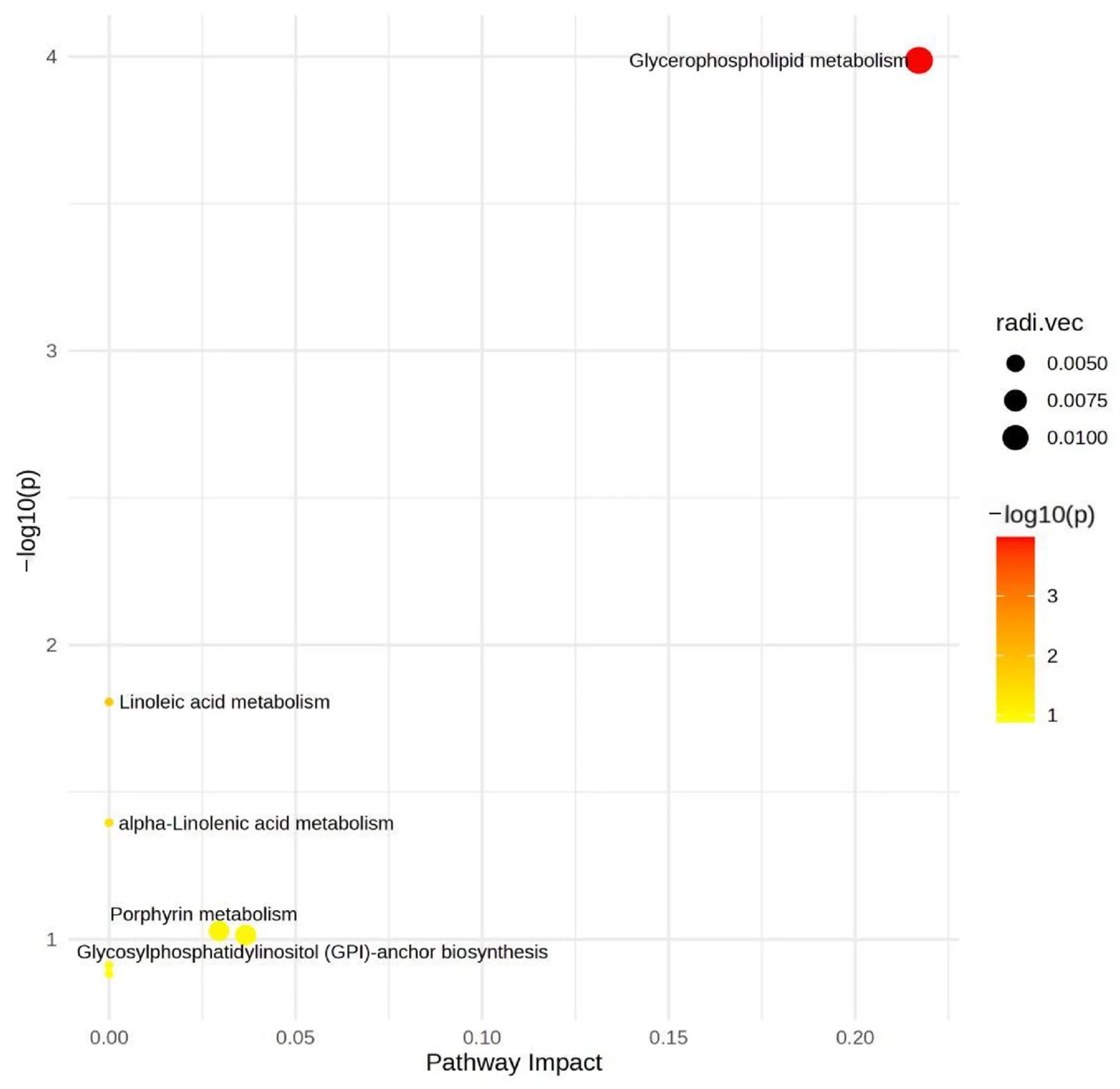
Pathway analysis of metabolites.

To explore the potential metabolic pathways associated with the aforementioned 12 differential features, metabolites matching the accurate mass (±10 ppm) were imported into MetaboAnalyst 6.0 for pathway enrichment analysis. A total of 7 metabolic pathways were identified, with only one reaching statistical significance (FDR_q < 0.05): glycerophospholipid metabolism (Figure 2 and Table S4). The remaining 6 pathways (linoleic acid metabolism, alpha-linolenic acid metabolism, porphyrin metabolism, glycosylphosphatidylinositol (GPI)-anchor biosynthesis, steroid biosynthesis, and arachidonic acid metabolism) each had only a single hit, likely representing single-metabolite-driven effects and failing to reach the significance threshold (FDR_q > 0.05). These pathway analysis results should be considered an exploratory reference only, as they may be subject to limitations, including single-hit constraints, uncertainty in putative annotations, and database mapping artifacts.

## 4. Discussion

In this study, LC–MS was used to analyze plasma samples from 143 workers exposed to and not exposed to occupational stress, and a total of 709 features were detected in positive-ion mode. After feature selection, 12 differentially abundant metabolite features were ultimately identified, including one that was positively correlated and 11 that were negatively correlated with occupational stress. ROC curve analysis showed that five features (M573T1, M264T3, M673T5, M686T27, and M569T1) could serve as preliminary indicators of occupational stress. Pathway analysis showed an association between occupational stress and glycerophospholipid metabolism.

In this study, 12 differential features were significantly associated with occupational stress. Putative annotation (MSI level 3) indicated that these features mainly involved lipid metabolites, including diacylglycerols (DGs), cholesteryl esters (CEs), lysophospholipids (LysoPC, LysoPE), phospholipids (PE/PC), and ceramides. Numerous previous studies have also confirmed a significant association between occupational stress exposure and lipid metabolism disorders [25]. One feature, M687T1, was positively correlated with occupational stress and corresponded to metabolites, including DGs and CEs. The remaining 11 features negatively correlated with occupational stress included mainly lysophosphatidylcholines, various dipeptides/γ-glutamyl peptides (e.g., His-Thr, Glu-Leu, γ-glutamyl ornithine), long-chain acylcarnitines (arachidonyl carnitine), porphyrins (coproporphyrin III), conjugated bile acids, and some DGs/CEs. This pattern indicates that occupational stress may cause marked membrane lipid remodeling and polyunsaturated fatty acid (PUFA) metabolism disorders. This result is consistent with the findings of Butler et al., who reported reduced circulating triglyceride levels following acute stressor exposure [26]. In addition, other studies have indicated that elevated levels of lipid metabolites such as triglycerides are associated with an increased risk of subsequently developing depression, anxiety, and stress-related disorders [13]. However, lipid levels are influenced by multiple factors, such as individual dietary habits; therefore, caution is warranted when using lipid levels as a measure of occupational stress.

Furthermore, we obtained deeper insights into the potential metabolic pathways underlying the response to occupational stress. Pathway enrichment analysis revealed that 12 differential features were significantly enriched in the glycerophospholipid metabolism pathway, providing clues for elucidating the mechanisms underlying the effects of occupational stress. Glycerophospholipids are not only core components of cell membranes but also serve as reservoirs for numerous polyunsaturated fatty acids, such as cholesteryl linoleate and arachidonyl carnitine. Chronic occupational stress can drive membrane phospholipid remodeling through multiple pathways. Previous studies have shown that chronic occupational stress elevates ceramide (Cer) levels while reducing phosphatidylethanolamine (PE) levels [27]. Moreover, Ungur et al., in their study of burnout among healthcare workers, reported decreased plasma phosphatidylcholine (PC) levels in the high-burnout group [28]. These findings are consistent with the results of the present study. In addition, occupational stress is closely linked to psychological status; individuals under higher levels of work stress are more prone to severe depressive and anxiety symptoms [29]. Extensive evidence supports a role for glycerophospholipid metabolism in the onset and development of depression. For instance, one study highlighted significant disturbances in the glycerophospholipid metabolism pathway among patients with major depressive disorder (MDD) [30]. Furthermore, animal studies of chronic mild stress have confirmed that chronic mild pressure alters glycerophospholipid metabolism in rats and induces depression-like behaviors [31]. However, the in vivo metabolic network is highly complex, and the glycerophospholipid pathway extensively interacts with other lipid metabolic processes (e.g., sphingolipid metabolism and fatty acid oxidation). Further studies are needed to identify occupational stress-specific biomarkers and clarify their causal temporal relationships.

In this study, we provide preliminary evidence for the metabolic characteristics of individuals exposed to occupational stress and identify the glycerophospholipid metabolism pathway as a key pathway significantly associated with occupational stress exposure. Several limitations should be noted. First, the cross-sectional design, relatively small sample size, exclusively male population, and single geographic location preclude causal or temporal inference. Further research utilizing a larger cohort is needed in the future. Second, job stress was assessed using the self-reported CJCQ, which may have introduced subjective bias. Moreover, despite adjustment for shift work, smoking, alcohol consumption, and exercise, residual confounding may persist due to long-term dietary habits, supplement use, sleep quality, psychological symptoms, physical activity, and other occupational exposures. Although factory-provided meals may have reduced dietary variation among workers, their potential influence cannot be excluded. Third, standard substances and MS/MS were not used in the identification of metabolites. Therefore, these findings are hypothesis-generating and require future validation through independent cohort studies.

## 5. Conclusions

In this study, a plasma metabolomics approach based on untargeted LC–MS was employed to investigate the biochemical characteristics of occupational stress exposure in manufacturing workers. A total of 12 stress-associated characteristics and candidate discriminatory features were identified, among which 5 features exhibited moderate discriminatory ability and corresponded mainly to lipid metabolites. Pathway enrichment analysis indicated that the glycerophospholipid metabolism pathway was the primary perturbed metabolic pathway. In summary, these findings suggest that occupational stress is associated with systemic disturbances in lipid metabolism and membrane-related signaling pathways. As this was a cross-sectional study, while the observed metabolic alterations may be linked to stress-related diseases, further research is needed for validation. This study provides a potential foundation for future biomarker validation and mechanistic research in the field of occupational health.

## Figures and Tables

**Table 1:** Gradient program.

Time (min)	Flow Rate (mL/min)	A (%)	B (%)	Pressure (bar)
0.00	0.25	98.0	2.0	400.0
9.00	0.25	40.0	60.0	400.0
18.00	0.25	40.0	60.0	400.0
20.00	0.25	0.0	100.0	400.0
30.00	0.25	0.0	100.0	400.0

**Table 2:** Column washing and re-equilibration program.

Time (min)	Flow Rate (mL/min)	A (%)	B (%)	Pressure (bar)
0.0	0.25	2.0	98.0	400.0
7.0	0.25	2.0	98.0	400.0
14.0	0.25	98.0	2.0	400.0

**Table 3:** Mass spectrometric parameters.

Parameter	Positive Mode	Negative Mode
Acquisition mode	Full-scan MS	Full-scan MS
Mass range (*m*/*z*)	50–1500	50–1500
Capillary voltage (VCap)	3000 V	3000 V
Fragmentor voltage	70 V	70 V
Drying gas temperature	350 °C	350 °C
Drying gas flow	8.0 L/min	8.0 L/min
Nebulizer pressure	35 psig	35 psig
Collision energy	Not applicable	Not applicable

**Table 4:** Demographic characteristics of job stress group and non-job stress group.

Variable	N (*n* = 143)	Job-Stress (*n* = 78)	Non Job Stress (*n* = 65)	Student-*t*/*χ^2^*	*p* Value
BMI (kg/m^2^) mean ± SD	22.98 ± 3.27	22.75 ± 3.33	23.26 ± 3.20	−0.93	0.355
Height (cm) mean ± SD	169.20 ± 7.41	169.13 ± 7.49	169.28 ± 7.37	−0.13	0.901
Weight (kg) mean ± SD	65.72 ± 9.92	65.09 ± 10.70	66.48 ± 8.92	−0.84	0.404
CJCQ score mean ± SD	1.08 ± 0.30	1.27 ± 0.28	0.86 ± 0.12		
Education level				- ^b^	0.619
College polytechnic school and below	139	76	63		
bachelor degree and above	4	2	2		
Occupational tenure				10.47	0.013 ^a^
1–5 years	87	46	41		
5–10 years	30	21	9		
10–15 years	14	9	5		
>15 years	12	2	10		
Shift status				1.27 ^c^	0.955
Never	48	24	24		
<5 times/month	8	4	4		
5–10 times/month	21	11	10		
10–15 times/month	40	24	16		
15–20 times/month	16	9	7		
>20 times/month	10	6	4		
Smoking consumption				1.93	0.387
0 per day	78	43	35		
<10 per day	49	24	25		
≥10 per day	16	11	5		
Alcohol consumption				1.13 ^c^	0.814
0 times/week	95	52	43		
1–2 times/week	39	21	18		
2–3 times/week	4	3	1		
>3 times/week	5	2	3		
Exercise				0.48	0.794
0 times/week	17	10	7		
1–2 times/week	113	60	53		
>2 times/week	13	8	5		

Note: ^a^ *p* < 0.05 indicates a statistically significant difference between the job stress group and the non-job stress group. ^b^ Fisher’s exact test or ^c^ Fisher-Freeman-Halton exact test were used when more than 20% of the cells in the contingency table had an expected count less than 5.

**Table 5:** Correlation analysis between differential features and job stress.

Feature Id	*m*/z	Retention Time	FDR_q	Correlation	Ionization Mode
M673T5	672.6	5.48	<0.001	−0.344	positive
M615T1	614.6	0.79	<0.001	−0.292	positive
M341T2	341.2	2.50	<0.001	−0.355	positive
M525T1	525.4	0.80	<0.001	−0.279	positive
M573T1	572.6	0.83	<0.001	−0.430	positive
M264T3	264.1	2.54	<0.001	−0.352	positive
M686T27	686.4	27.44	<0.001	−0.308	positive
M589T2	588.6	2.05	<0.001	−0.361	positive
M659T1	658.6	0.78	<0.001	−0.272	positive
M715T3	714.6	2.96	<0.001	−0.333	positive
M569T1	569.5	0.79	0.002	−0.250	positive
M687T1	686.7	0.84	<0.001	0.319	positive

Note: The full model was adjusted for BMI, education level, occupational tenure, shift status, smoking consumption, alcohol consumption, and exercise. Categorical covariates were incorporated into the model as dummy variables using n-1 coding, and the group with the lowest risk was set as the reference category for each variable to ensure the interpretability of effect size estimates.

## Data Availability

The data presented in this study are available on request from the corresponding author. The data are not publicly available due to privacy restrictions.

## Supplementary Materials

The following supporting information can be downloaded at https://www.mdpi.com/article/10.3390/metabo16100790/s1. Figure S1. Feature count flowchart of metabolomics data preprocessing; Figure S2. Extracted ion chromatograms (EICs) of plasma samples showing peak cluster patterns. (A) Concentrated peak cluster. (B) Dispersed peak cluster; Figure S3. OPLS-DA scores of the job stress group and non-job stress group under positive and negative ion modes (A: positive ion mode, B: negative ion mode) and C: 200-permutation test chart of the job stress group and non-job stress group under positive ion mode. Table S1. Initial screening of 23 differential features in positive ion mode; Table S2. Twelve putatively annotated differential features corresponding to metabolites; Table S3. Apparent area under the receiver operating characteristic curves (AUCs) of the 12 differential features; Table S4. Metabolic pathway analysis.

## Abbreviations

The following abbreviations are used in this manuscript:

LC–MS	liquid chromatography–mass spectrometry
CJCQ	The Chinese Job Content Questionnaire
AUC	area under the curve
QC	Quality control
RSD	relative standard deviation
ROC	receiver operating characteristic curves
OPLS-DA	orthogonal partial least squares discriminant analysis
EIC	extracted ion chromatogram
MCCV	Monte Carlo cross-validation
