# Supplementary material for: Untargeted Plasma Metabolomics Reveals Metabolic Differences Associated with Job Stress in Manufacturing Workers: A Cross-Sectional Study
Source: Metabolites. 2026 Oct 18;16(10):790. doi: 10.3390/metabo16100790 (PMC13707163; doi:10.3390/metabo16100790)
Supplement: Supplementary file 1 [file metabolites-16-00790-s001.zip › metabolites-4479318-supplementary-Figure S2.pdf]

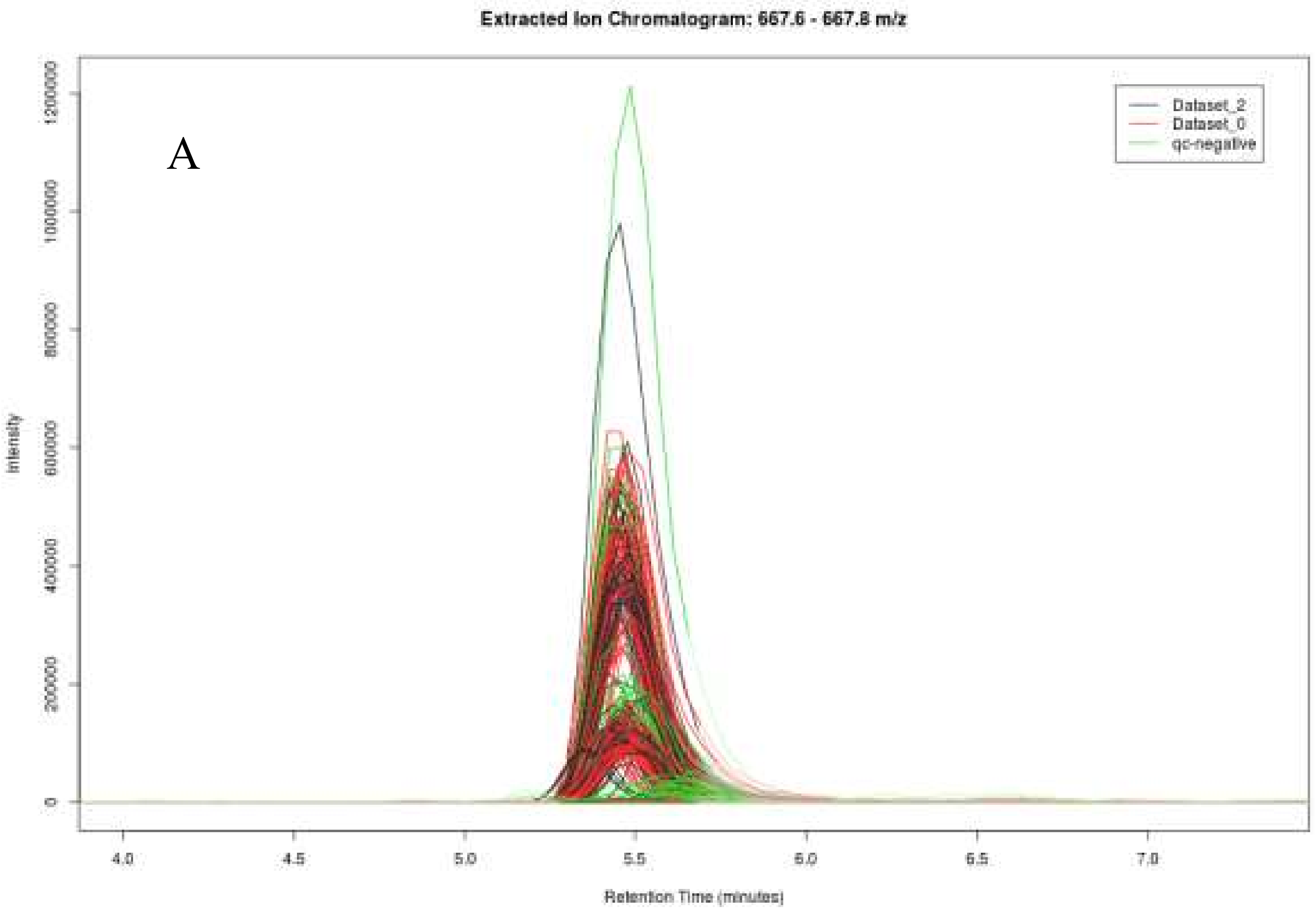

Extracted Ion Chromatogram: 667.6 - 667.8 m/z
A
Dataset_2
Dataset_0
qc-negative
Intensity
0
200000
400000
600000
800000
1000000
1200000
4.0
4.5
5.0
5.5
6.0
6.5
7.0
Retention Time (minutes)

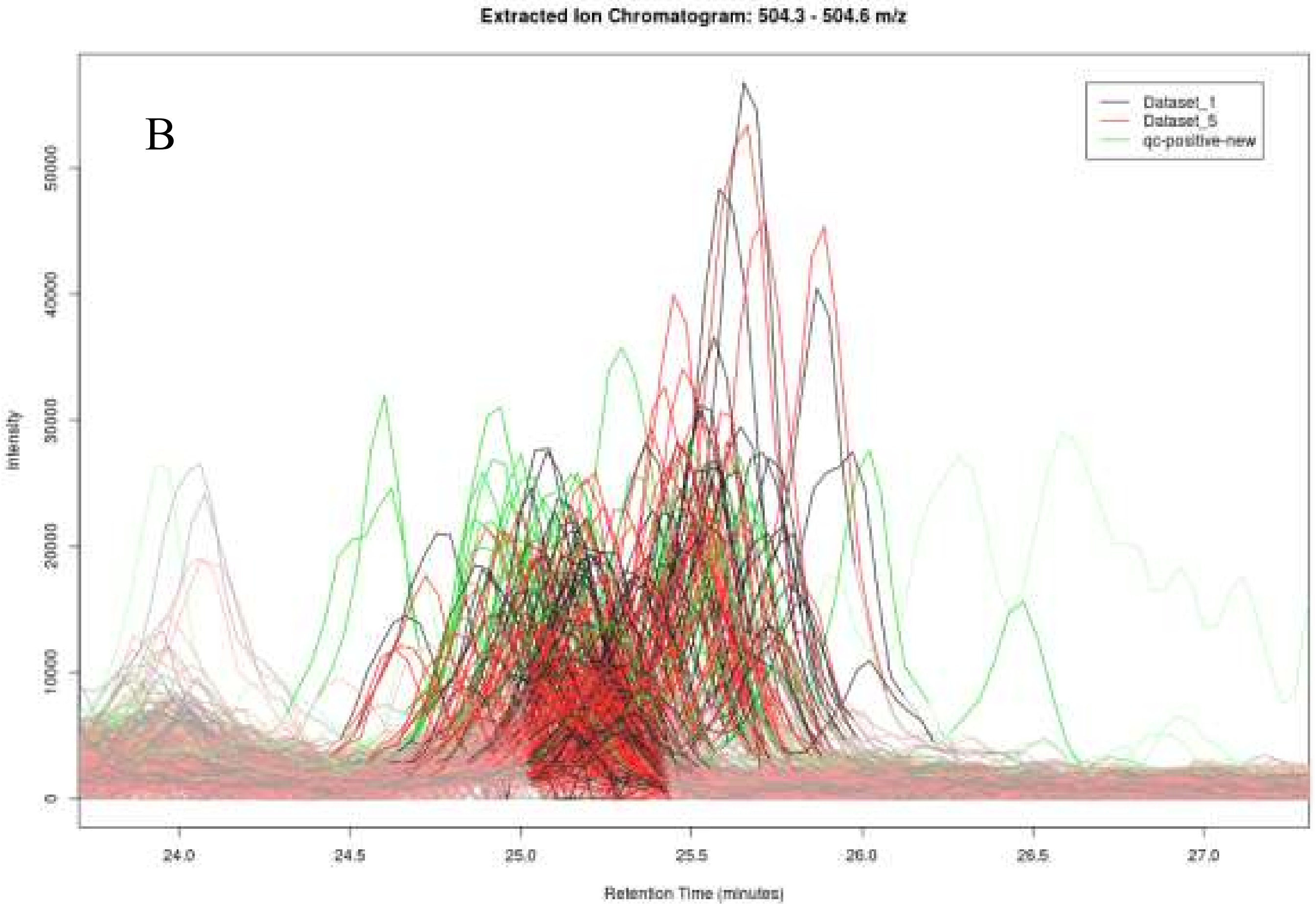

Figure S2. Extracted ion chromatograms (EICs) of plasma samples showing peak cluster patterns. (A) Concentrated peak cluster. (B) Dispersed peak cluster.
