# Supplementary material for: Untargeted Plasma Metabolomics Reveals Metabolic Differences Associated with Job Stress in Manufacturing Workers: A Cross-Sectional Study
Source: Metabolites. 2026 Oct 18;16(10):790. doi: 10.3390/metabo16100790 (PMC13707163; doi:10.3390/metabo16100790)
Supplement: Supplementary file 1 [file metabolites-16-00790-s001.zip › metabolites-4479318-supplementary-Figure S3.pdf]

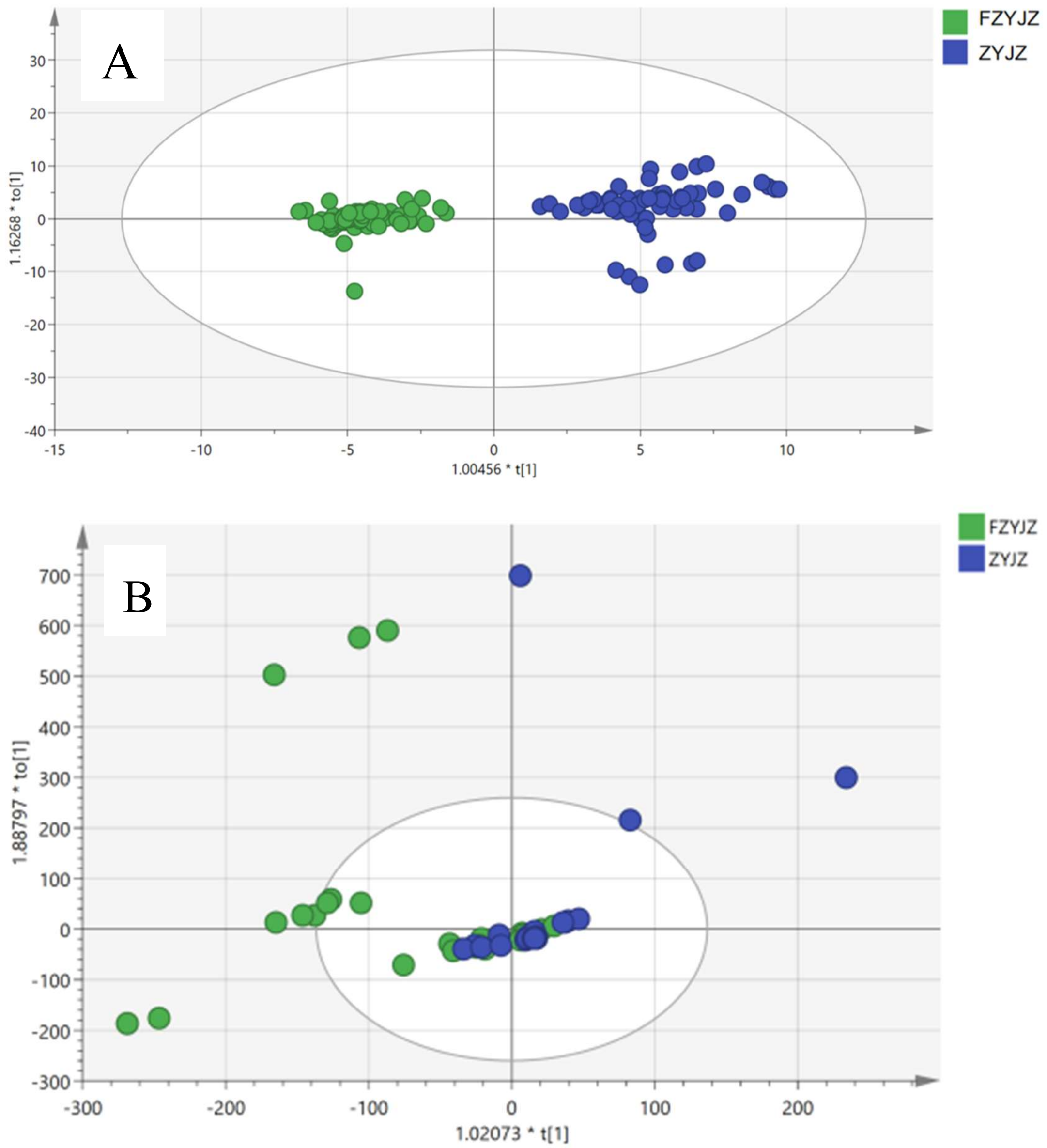
A
FZYJZ
ZYJZ
1.16268 * to[1]
1.00456 * t[1]
B
FZYJZ
ZYJZ
1.88797 * to[1]
1.02073 * t[1]

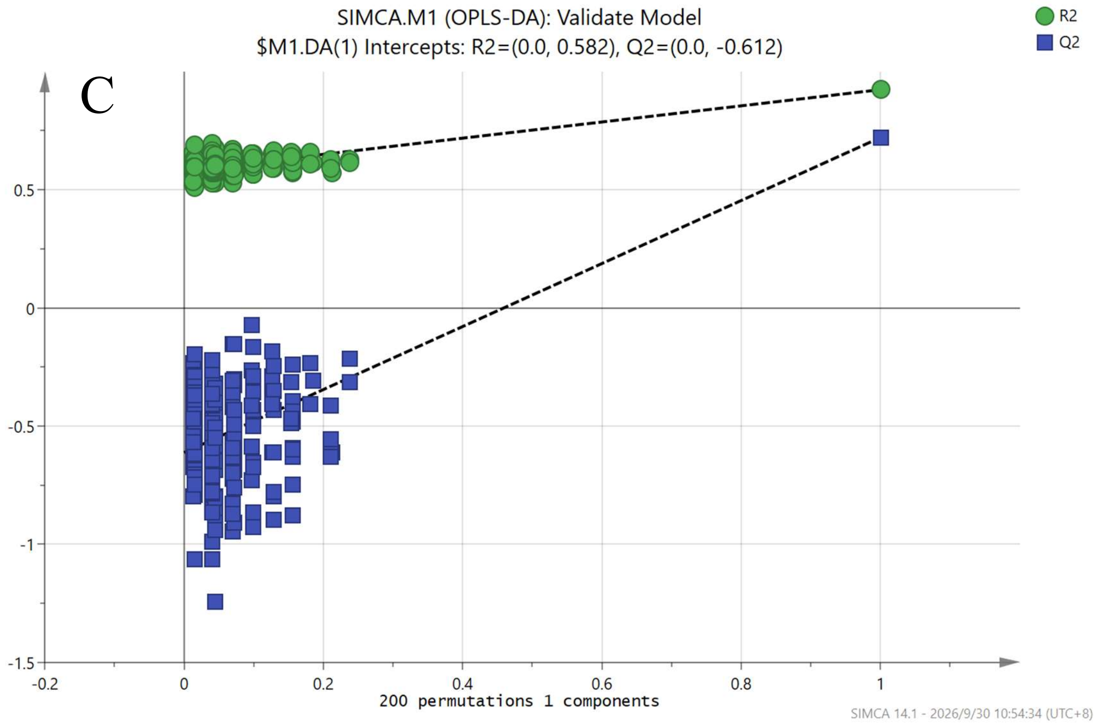

Figure S3. OPLS-DA scores of job stress group and non-job stress group under positive and negative ion modes (A: positive ion mode, B: negative ion mode) and C: 200-permutation test chart of job stress group and non-job stress group under positive ion mode.
