# Supplementary material for: Untargeted Plasma Metabolomics Reveals Metabolic Differences Associated with Job Stress in Manufacturing Workers: A Cross-Sectional Study
Source: Metabolites. 2026 Oct 18;16(10):790. doi: 10.3390/metabo16100790 (PMC13707163; doi:10.3390/metabo16100790)
Supplement: Supplementary file 1 [file metabolites-16-00790-s001.zip › metabolites-4479318-supplementary-Table S1.pdf]

Table S1. Initial screening of 23 differential features in positive ion mode.

Feature id	*P*	FDR_q	VIP
M525T1	0.0004	0.0286	1.362
M569T1	0.0014	0.0442	1.324
M1279T3	0.0011	0.0374	1.879
M1257T3	0.0000	0.0006	1.763
M615T1	0.0003	0.0233	1.469
M589T2	0.0010	0.0374	1.273
M673T5	0.0000	0.0006	1.468
M715T3	0.0012	0.0391	1.185
M341T2	0.0004	0.0286	1.386
M686T27	0.0009	0.0374	1.383
M158T3	0.0008	0.0374	1.275
M1326T3	0.0000	0.0006	1.547
M1254T3	0.0000	0.0007	1.838
M360T5	0.0011	0.0374	1.331
M1328T3	0.0000	0.0010	1.534
M1256T3	0.0000	0.0024	1.570
M659T1	0.0010	0.0374	1.282
M1488T3	0.0007	0.0348	1.297
M264T3	0.0008	0.0374	1.417
M573T1	0.0005	0.0299	1.809
M799T1	0.0000	0.0004	1.942
M687T1	0.0000	0.0024	1.593

M257T1	0.0006	0.0339	1.221
