# Supplementary material for: Untargeted Plasma Metabolomics Reveals Metabolic Differences Associated with Job Stress in Manufacturing Workers: A Cross-Sectional Study
Source: Metabolites. 2026 Oct 18;16(10):790. doi: 10.3390/metabo16100790 (PMC13707163; doi:10.3390/metabo16100790)
Supplement: Supplementary file 1 [file metabolites-16-00790-s001.zip › metabolites-4479318-supplementary-Table S2.pdf]

Table S2. Twelve putatively annotated differential features corresponding to metabolites

| Feature id | m/z | retention time | metabolite | HMDB |
|---|---|---|---|---|
| M673T5 | 672.6 | 5.48 | CE(16:1(9Z)) | HMDB00658 |
| M673T5 | 672.6 | 5.48 | DG(14:1(9Z)/24:1(15Z)/0:0) | HMDB07065 |
| M673T5 | 672.6 | 5.48 | DG(18:1(11Z)/20:1(11Z)/0:0) | HMDB07195 |
| M673T5 | 672.6 | 5.48 | DG(20:0/18:2(9Z,12Z)/0:0) | HMDB07364 |
| M673T5 | 672.6 | 5.48 | DG(20:1(11Z)/18:1(11Z)/0:0) | HMDB07391 |
| M673T5 | 672.6 | 5.48 | DG(20:1(11Z)/18:1(9Z)/0:0) | HMDB07392 |
| M673T5 | 672.6 | 5.48 | DG(20:2(11Z,14Z)/18:0/0:0) | HMDB07419 |
| M673T5 | 672.6 | 5.48 | DG(22:1(13Z)/16:1(9Z)/0:0) | HMDB07621 |
| M673T5 | 672.6 | 5.48 | DG(22:2(13Z,16Z)/16:0/0:0) | HMDB07649 |
| M673T5 | 672.6 | 5.48 | DG(24:1(15Z)/14:1(9Z)/0:0) | HMDB07821 |
| M673T5 | 672.6 | 5.48 | DG(18:0/20:3(5Z,8Z,11Z)/0:0) | HMDB07168 |
| M673T5 | 672.6 | 5.48 | DG(18:1(11Z)/20:2(11Z,14Z)/0:0) | HMDB07196 |

| | | | | |
|---|---|---|---|---|
| M673T5 | 672.6 | 5.48 | DG(18:3(6Z,9Z,12Z)/20:0/0:0) | HMDB07281 |
| M673T5 | 672.6 | 5.48 | DG(20:0/18:3(6Z,9Z,12Z)/0:0) | HMDB07365 |
| M673T5 | 672.6 | 5.48 | DG(20:0/18:3(9Z,12Z,15Z)/0:0) | HMDB07366 |
| M673T5 | 672.6 | 5.48 | DG(20:1(11Z)/18:2(9Z,12Z)/0:0) | HMDB07393 |
| M673T5 | 672.6 | 5.48 | DG(20:2(11Z,14Z)/18:1(11Z)/0:0) | HMDB07420 |
| M673T5 | 672.6 | 5.48 | DG(20:2(11Z,14Z)/18:1(9Z)/0:0) | HMDB07421 |
| M673T5 | 672.6 | 5.48 | DG(20:3(5Z,8Z,11Z)/18:0/0:0) | HMDB07448 |
| M673T5 | 672.6 | 5.48 | DG(20:3(8Z,11Z,14Z)/18:0/0:0) | HMDB07477 |
| M673T5 | 672.6 | 5.48 | DG(22:2(13Z,16Z)/16:1(9Z)/0:0) | HMDB07650 |
| M673T5 | 672.6 | 5.48 | CE(19:0) | HMDB06738 |
| M687T1 | 686.7 | 0.84 | DG(14:0/24:0/0:0) | HMDB07035 |
| M687T1 | 686.7 | 0.84 | DG(22:0/16:0/0:0) | HMDB07591 |
| M687T1 | 686.7 | 0.84 | DG(24:0/14:0/0:0) | HMDB07791 |
| M687T1 | 686.7 | 0.84 | Cholesteryl linoleate | HMDB00610 |

| | | | | |
|---|---|---|---|---|
| M687T1 | 686.7 | 0.84 | CE(18:1) | HMDB00918 |
| M687T1 | 686.7 | 0.84 | CE(18:1(11Z)) | HMDB05189 |
| M687T1 | 686.7 | 0.84 | Cer(d18:0/23:0) | HMDB11767 |
| M615T1 | 614.6 | 0.79 | DG(14:0/20:2(11Z,14Z)/0:0) | HMDB07022 |
| M615T1 | 614.6 | 0.79 | DG(14:1(9Z)/20:1(11Z)/0:0) | HMDB07050 |
| M615T1 | 614.6 | 0.79 | DG(16:1(9Z)/18:1(11Z)/0:0) | HMDB07130 |
| M615T1 | 614.6 | 0.79 | DG(18:1(11Z)/16:1(9Z)/0:0) | HMDB07186 |
| M615T1 | 614.6 | 0.79 | DG(18:1(9Z)/16:1(9Z)/0:0) | HMDB07215 |
| M615T1 | 614.6 | 0.79 | DG(18:2(9Z,12Z)/16:0/0:0) | HMDB07243 |
| M615T1 | 614.6 | 0.79 | DG(20:1(11Z)/14:1(9Z)/0:0) | HMDB07386 |
| M615T1 | 614.6 | 0.79 | DG(20:2(11Z,14Z)/14:0/0:0) | HMDB07414 |
| M341T2 | 341.2 | 2.50 | Octadecanedioic acid | HMDB00782 |
| M341T2 | 341.2 | 2.50 | 9,10-DiHOME | HMDB04704 |
| M341T2 | 341.2 | 2.50 | 12,13-DiHOME | HMDB04705 |

| | | | | |
|---|---|---|---|---|
| M341T2 | 341.2 | 2.50 | 5-Androstene-3b,16b,17a-triol | HMDB00523 |
| M341T2 | 341.2 | 2.50 | 11-Hydroxyandrosterone | HMDB02984 |
| M341T2 | 341.2 | 2.50 | 5-Androstene-3b,16a,17a-triol | HMDB00540 |
| M525T1 | 525.4 | 0.80 | LysoPE(0:0/20:1(11Z)) | HMDB11482 |
| M525T1 | 525.4 | 0.80 | LysoPE(20:1(11Z)/0:0) | HMDB11512 |
| M525T1 | 525.4 | 0.80 | LysoPC(P-16:0) | HMDB10407 |
| M573T1 | 572.6 | 0.83 | Cer(d18:0/18:1(11Z)) | HMDB11762 |
| M573T1 | 572.6 | 0.83 | DG(14:0/16:1(9Z)/0:0) | HMDB07012 |
| M573T1 | 572.6 | 0.83 | DG(14:1(9Z)/16:0/0:0) | HMDB07040 |
| M573T1 | 572.6 | 0.83 | DG(16:0/14:1(9Z)/0:0) | HMDB07096 |
| M573T1 | 572.6 | 0.83 | DG(16:1(9Z)/14:0/0:0) | HMDB07124 |
| M264T3 | 264.1 | 2.54 | Histidinyl-Threonine | HMDB28895 |
| M264T3 | 264.1 | 2.54 | Threoninyl-Histidine | HMDB29063 |
| M264T3 | 264.1 | 2.54 | Glutamyl-Isoleucine | HMDB28822 |

| | | | | |
|---|---|---|---|---|
| M264T3 | 264.1 | 2.54 | Glutamyl-Leucine | HMDB28823 |
| M264T3 | 264.1 | 2.54 | Isoleucyl-Glutamate | HMDB28906 |
| M264T3 | 264.1 | 2.54 | Leucyl-Glutamate | HMDB28928 |
| M264T3 | 264.1 | 2.54 | γ glutamyl ornithine | HMDB02248 |
| M264T3 | 264.1 | 2.54 | Alpha-Aspartyl-lysine | HMDB04987 |
| M264T3 | 264.1 | 2.54 | Aspartyl-Lysine | HMDB28758 |
| M264T3 | 264.1 | 2.54 | Lysyl-Aspartate | HMDB28947 |
| M686T27 | 686.4 | 27.44 | (3a,5b,7b)-24-[(carboxymethyl)amino]-7-hydroxy-24-oxocholan-3-yl -b-D-glucopyranosiduronic acid, | HMDB02618 |
| M686T27 | 686.4 | 27.44 | Coproporphyrin III | HMDB00570 |
| M589T2 | 588.6 | 2.05 | DG(14:0/18:2(9Z,12Z)/0:0) | HMDB07016 |
| M589T2 | 588.6 | 2.05 | DG(14:1(9Z)/18:1(11Z)/0:0) | HMDB07043 |
| M589T2 | 588.6 | 2.05 | DG(14:1(9Z)/18:1(9Z)/0:0) | HMDB07044 |
| M589T2 | 588.6 | 2.05 | DG(18:1(11Z)/14:1(9Z)/0:0) | HMDB07183 |
| M589T2 | 588.6 | 2.05 | DG(18:1(9Z)/14:1(9Z)/0:0) | HMDB07212 |

| | | | | |
|---|---|---|---|---|
| M589T2 | 588.6 | 2.05 | DG(18:2(9Z,12Z)/14:0/0:0) | HMDB07240 |
| M589T2 | 588.6 | 2.05 | DG(14:0/18:3(6Z,9Z,12Z)/0:0) | HMDB07017 |
| M589T2 | 588.6 | 2.05 | DG(14:0/18:3(9Z,12Z,15Z)/0:0) | HMDB07018 |
| M589T2 | 588.6 | 2.05 | DG(14:1(9Z)/18:2(9Z,12Z)/0:0) | HMDB07045 |
| M589T2 | 588.6 | 2.05 | DG(18:2(9Z,12Z)/14:1(9Z)/0:0) | HMDB07241 |
| M589T2 | 588.6 | 2.05 | DG(18:3(6Z,9Z,12Z)/14:0/0:0) | HMDB07269 |
| M589T2 | 588.6 | 2.05 | DG(18:3(9Z,12Z,15Z)/14:0/0:0) | HMDB07298 |
| M659T1 | 658.6 | 0.78 | DG(14:0/22:2(13Z,16Z)/0:0) | HMDB07030 |
| M659T1 | 658.6 | 0.78 | DG(14:1(9Z)/22:1(13Z)/0:0) | HMDB07058 |
| M659T1 | 658.6 | 0.78 | DG(18:1(11Z)/18:1(11Z)/0:0) | HMDB07188 |
| M659T1 | 658.6 | 0.78 | DG(18:1(11Z)/18:1(9Z)/0:0) | HMDB07189 |
| M659T1 | 658.6 | 0.78 | DG(18:1(9Z)/18:1(11Z)/0:0) | HMDB07217 |
| M659T1 | 658.6 | 0.78 | DG(18:2(9Z,12Z)/18:0/0:0) | HMDB07245 |
| M659T1 | 658.6 | 0.78 | DG(20:1(11Z)/16:1(9Z)/0:0) | HMDB07389 |

| | | | | |
|---|---|---|---|---|
| M659T1 | 658.6 | 0.78 | DG(20:2(11Z,14Z)/16:0/0:0) | HMDB07417 |
| M659T1 | 658.6 | 0.78 | DG(22:1(13Z)/14:1(9Z)/0:0) | HMDB07618 |
| M659T1 | 658.6 | 0.78 | DG(22:2(13Z,16Z)/14:0/0:0) | HMDB07646 |
| M659T1 | 658.6 | 0.78 | DG(14:0/20:0/0:0) | HMDB07020 |
| M659T1 | 658.6 | 0.78 | DG(20:0/14:0/0:0) | HMDB07356 |
| M659T1 | 658.6 | 0.78 | DG(16:0/22:5(4Z,7Z,10Z,13Z,16Z)/0:0) | HMDB07119 |
| M659T1 | 658.6 | 0.78 | DG(18:1(11Z)/20:4(5Z,8Z,11Z,14Z)/0:0) | HMDB07199 |
| M659T1 | 658.6 | 0.78 | DG(18:1(11Z)/20:4(8Z,11Z,14Z,17Z)/0:0) | HMDB07200 |
| M659T1 | 658.6 | 0.78 | DG(18:1(9Z)/20:4(8Z,11Z,14Z,17Z)/0:0) | HMDB07229 |
| M659T1 | 658.6 | 0.78 | DG(18:2(9Z,12Z)/20:3(5Z,8Z,11Z)/0:0) | HMDB07255 |
| M659T1 | 658.6 | 0.78 | DG(18:3(6Z,9Z,12Z)/20:2(11Z,14Z)/0:0) | HMDB07283 |
| M659T1 | 658.6 | 0.78 | DG(18:4(6Z,9Z,12Z,15Z)/20:1(11Z)/0:0) | HMDB07340 |
| M659T1 | 658.6 | 0.78 | DG(20:1(11Z)/18:4(6Z,9Z,12Z,15Z)/0:0) | HMDB07396 |
| M659T1 | 658.6 | 0.78 | DG(20:2(11Z,14Z)/18:3(6Z,9Z,12Z)/0:0) | HMDB07423 |

| | | | | |
|---|---|---|---|---|
| M659T1 | 658.6 | 0.78 | DG(20:2(11Z,14Z)/18:3(9Z,12Z,15Z)/0:0) | HMDB07424 |
| M659T1 | 658.6 | 0.78 | DG(20:3(5Z,8Z,11Z)/18:2(9Z,12Z)/0:0) | HMDB07451 |
| M659T1 | 658.6 | 0.78 | DG(20:3(8Z,11Z,14Z)/18:2(9Z,12Z)/0:0) | HMDB07480 |
| M659T1 | 658.6 | 0.78 | DG(20:4(5Z,8Z,11Z,14Z)/18:1(11Z)/0:0) | HMDB07507 |
| M659T1 | 658.6 | 0.78 | DG(20:4(5Z,8Z,11Z,14Z)/18:1(9Z)/0:0) | HMDB07508 |
| M659T1 | 658.6 | 0.78 | DG(20:4(8Z,11Z,14Z,17Z)/18:1(11Z)/0:0) | HMDB07536 |
| M659T1 | 658.6 | 0.78 | DG(20:4(8Z,11Z,14Z,17Z)/18:1(9Z)/0:0) | HMDB07537 |
| M659T1 | 658.6 | 0.78 | DG(20:5(5Z,8Z,11Z,14Z,17Z)/18:0/0:0) | HMDB07564 |
| M659T1 | 658.6 | 0.78 | DG(22:4(7Z,10Z,13Z,16Z)/16:1(9Z)/0:0) | HMDB07679 |
| M659T1 | 658.6 | 0.78 | DG(22:5(4Z,7Z,10Z,13Z,16Z)/16:0/0:0) | HMDB07707 |
| M659T1 | 658.6 | 0.78 | DG(22:5(7Z,10Z,13Z,16Z,19Z)/16:0/0:0) | HMDB07736 |
| M659T1 | 658.6 | 0.78 | DG(16:1(9Z)/22:5(4Z,7Z,10Z,13Z,16Z)/0:0) | HMDB07148 |
| M659T1 | 658.6 | 0.78 | DG(18:1(11Z)/20:5(5Z,8Z,11Z,14Z,17Z)/0:0) | HMDB07201 |
| M659T1 | 658.6 | 0.78 | DG(18:2(9Z,12Z)/20:4(8Z,11Z,14Z,17Z)/0:0) | HMDB07258 |

| | | | | |
|---|---|---|---|---|
| M659T1 | 658.6 | 0.78 | DG(18:3(6Z,9Z,12Z)/20:3(5Z,8Z,11Z)/0:0) | HMDB07284 |
| M659T1 | 658.6 | 0.78 | DG(18:3(6Z,9Z,12Z)/20:3(8Z,11Z,14Z)/0:0) | HMDB07285 |
| M659T1 | 658.6 | 0.78 | DG(18:3(9Z,12Z,15Z)/20:3(5Z,8Z,11Z)/0:0) | HMDB07313 |
| M659T1 | 658.6 | 0.78 | DG(18:4(6Z,9Z,12Z,15Z)/20:2(11Z,14Z)/0:0) | HMDB07341 |
| M659T1 | 658.6 | 0.78 | DG(20:2(11Z,14Z)/18:4(6Z,9Z,12Z,15Z)/0:0) | HMDB07425 |
| M659T1 | 658.6 | 0.78 | DG(20:3(5Z,8Z,11Z)/18:3(6Z,9Z,12Z)/0:0) | HMDB07452 |
| M659T1 | 658.6 | 0.78 | DG(20:3(5Z,8Z,11Z)/18:3(9Z,12Z,15Z)/0:0) | HMDB07453 |
| M659T1 | 658.6 | 0.78 | DG(20:3(8Z,11Z,14Z)/18:3(6Z,9Z,12Z)/0:0) | HMDB07481 |
| M659T1 | 658.6 | 0.78 | DG(20:3(8Z,11Z,14Z)/18:3(9Z,12Z,15Z)/0:0) | HMDB07482 |
| M659T1 | 658.6 | 0.78 | DG(20:4(5Z,8Z,11Z,14Z)/18:2(9Z,12Z)/0:0) | HMDB07509 |
| M659T1 | 658.6 | 0.78 | DG(20:4(8Z,11Z,14Z,17Z)/18:2(9Z,12Z)/0:0) | HMDB07538 |
| M659T1 | 658.6 | 0.78 | DG(20:5(5Z,8Z,11Z,14Z,17Z)/18:1(11Z)/0:0) | HMDB07565 |
| M659T1 | 658.6 | 0.78 | DG(20:5(5Z,8Z,11Z,14Z,17Z)/18:1(9Z)/0:0) | HMDB07566 |
| M659T1 | 658.6 | 0.78 | DG(22:5(4Z,7Z,10Z,13Z,16Z)/16:1(9Z)/0:0) | HMDB07708 |

| | | | | |
|---|---|---|---|---|
| M659T1 | 658.6 | 0.78 | DG(22:5(7Z,10Z,13Z,16Z,19Z)/16:1(9Z)/0:0) | HMDB07737 |
| M659T1 | 658.6 | 0.78 | DG(22:6(4Z,7Z,10Z,13Z,16Z,19Z)/16:0/0:0) | HMDB07765 |
| M659T1 | 658.6 | 0.78 | lysoPC(26:0) | HMDB29205 |
| M659T1 | 658.6 | 0.78 | DG(15:0/20:5(5Z,8Z,11Z,14Z,17Z)/0:0) | HMDB07085 |
| M659T1 | 658.6 | 0.78 | DG(20:5(5Z,8Z,11Z,14Z,17Z)/15:0/0:0) | HMDB07561 |
| M715T3 | 714.6 | 2.96 | lysoPC(28:0) | HMDB29206 |
| M715T3 | 714.6 | 2.96 | PC(14:0/15:0) | HMDB07868 |
| M715T3 | 714.6 | 2.96 | PC(15:0/14:0) | HMDB07932 |
| M715T3 | 714.6 | 2.96 | PE(14:0/18:0) | HMDB08826 |
| M715T3 | 714.6 | 2.96 | PE(18:0/14:0) | HMDB08986 |
| M715T3 | 714.6 | 2.96 | DG(16:0/22:5(4Z,7Z,10Z,13Z,16Z)/0:0) | HMDB07119 |
| M715T3 | 714.6 | 2.96 | DG(18:1(11Z)/20:4(5Z,8Z,11Z,14Z)/0:0) | HMDB07199 |
| M715T3 | 714.6 | 2.96 | DG(18:1(11Z)/20:4(8Z,11Z,14Z,17Z)/0:0) | HMDB07200 |
| M715T3 | 714.6 | 2.96 | DG(18:1(9Z)/20:4(8Z,11Z,14Z,17Z)/0:0) | HMDB07229 |

| | | | | |
|---|---|---|---|---|
| M715T3 | 714.6 | 2.96 | DG(18:2(9Z,12Z)/20:3(5Z,8Z,11Z)/0:0) | HMDB07255 |
| M715T3 | 714.6 | 2.96 | DG(18:3(6Z,9Z,12Z)/20:2(11Z,14Z)/0:0) | HMDB07283 |
| M715T3 | 714.6 | 2.96 | DG(18:4(6Z,9Z,12Z,15Z)/20:1(11Z)/0:0) | HMDB07340 |
| M715T3 | 714.6 | 2.96 | DG(20:1(11Z)/18:4(6Z,9Z,12Z,15Z)/0:0) | HMDB07396 |
| M715T3 | 714.6 | 2.96 | DG(20:2(11Z,14Z)/18:3(6Z,9Z,12Z)/0:0) | HMDB07423 |
| M715T3 | 714.6 | 2.96 | DG(20:2(11Z,14Z)/18:3(9Z,12Z,15Z)/0:0) | HMDB07424 |
| M715T3 | 714.6 | 2.96 | DG(20:3(5Z,8Z,11Z)/18:2(9Z,12Z)/0:0) | HMDB07451 |
| M715T3 | 714.6 | 2.96 | DG(20:3(8Z,11Z,14Z)/18:2(9Z,12Z)/0:0) | HMDB07480 |
| M715T3 | 714.6 | 2.96 | DG(20:4(5Z,8Z,11Z,14Z)/18:1(11Z)/0:0) | HMDB07507 |
| M715T3 | 714.6 | 2.96 | DG(20:4(5Z,8Z,11Z,14Z)/18:1(9Z)/0:0) | HMDB07508 |
| M715T3 | 714.6 | 2.96 | DG(20:4(8Z,11Z,14Z,17Z)/18:1(11Z)/0:0) | HMDB07536 |
| M715T3 | 714.6 | 2.96 | DG(20:4(8Z,11Z,14Z,17Z)/18:1(9Z)/0:0) | HMDB07537 |
| M715T3 | 714.6 | 2.96 | DG(20:5(5Z,8Z,11Z,14Z,17Z)/18:0/0:0) | HMDB07564 |
| M715T3 | 714.6 | 2.96 | DG(22:4(7Z,10Z,13Z,16Z)/16:1(9Z)/0:0) | HMDB07679 |

| | | | | |
|---|---|---|---|---|
| M715T3 | 714.6 | 2.96 | DG(22:5(4Z,7Z,10Z,13Z,16Z)/16:0/0:0) | HMDB07707 |
| M715T3 | 714.6 | 2.96 | DG(22:5(7Z,10Z,13Z,16Z,19Z)/16:0/0:0) | HMDB07736 |
| M715T3 | 714.6 | 2.96 | DG(18:1(11Z)/22:5(4Z,7Z,10Z,13Z,16Z)/0:0) | HMDB07206 |
| M715T3 | 714.6 | 2.96 | DG(18:1(11Z)/22:5(7Z,10Z,13Z,16Z,19Z)/0:0) | HMDB07207 |
| M715T3 | 714.6 | 2.96 | DG(18:1(9Z)/22:5(4Z,7Z,10Z,13Z,16Z)/0:0) | HMDB07235 |
| M715T3 | 714.6 | 2.96 | DG(18:1(11Z)/22:5(7Z,10Z,13Z,16Z,19Z)/0:0) | HMDB07207 |
| M715T3 | 714.6 | 2.96 | DG(18:1(9Z)/22:5(4Z,7Z,10Z,13Z,16Z)/0:0) | HMDB07235 |
| M715T3 | 714.6 | 2.96 | DG(18:4(6Z,9Z,12Z,15Z)/22:2(13Z,16Z)/0:0) | HMDB07349 |
| M715T3 | 714.6 | 2.96 | DG(20:2(11Z,14Z)/20:4(8Z,11Z,14Z,17Z)/0:0) | HMDB07432 |
| M715T3 | 714.6 | 2.96 | DG(20:3(5Z,8Z,11Z)/20:3(5Z,8Z,11Z)/0:0) | HMDB07458 |
| M715T3 | 714.6 | 2.96 | DG(20:3(5Z,8Z,11Z)/20:3(8Z,11Z,14Z)/0:0) | HMDB07459 |
| M715T3 | 714.6 | 2.96 | DG(20:3(8Z,11Z,14Z)/20:3(5Z,8Z,11Z)/0:0) | HMDB07487 |
| M715T3 | 714.6 | 2.96 | DG(20:4(5Z,8Z,11Z,14Z)/20:2(11Z,14Z)/0:0) | HMDB07515 |
| M715T3 | 714.6 | 2.96 | DG(20:4(8Z,11Z,14Z,17Z)/20:2(11Z,14Z)/0:0) | HMDB07544 |

| | | | | |
|---|---|---|---|---|
| M715T3 | 714.6 | 2.96 | DG(20:5(5Z,8Z,11Z,14Z,17Z)/20:1(11Z)/0:0) | HMDB07572 |
| M715T3 | 714.6 | 2.96 | DG(22:2(13Z,16Z)/18:4(6Z,9Z,12Z,15Z)/0:0) | HMDB07657 |
| M715T3 | 714.6 | 2.96 | DG(22:4(7Z,10Z,13Z,16Z)/18:2(9Z,12Z)/0:0) | HMDB07683 |
| M715T3 | 714.6 | 2.96 | DG(22:5(4Z,7Z,10Z,13Z,16Z)/18:1(11Z)/0:0) | HMDB07710 |
| M715T3 | 714.6 | 2.96 | DG(22:5(4Z,7Z,10Z,13Z,16Z)/18:1(9Z)/0:0) | HMDB07711 |
| M715T3 | 714.6 | 2.96 | DG(22:5(7Z,10Z,13Z,16Z,19Z)/18:1(11Z)/0:0) | HMDB07739 |
| M715T3 | 714.6 | 2.96 | DG(22:5(7Z,10Z,13Z,16Z,19Z)/18:1(9Z)/0:0) | HMDB07740 |
| M715T3 | 714.6 | 2.96 | DG(22:6(4Z,7Z,10Z,13Z,16Z,19Z)/18:0/0:0) | HMDB07767 |
| M715T3 | 714.6 | 2.96 | DG(18:1(11Z)/22:6(4Z,7Z,10Z,13Z,16Z,19Z)/0:0) | HMDB07208 |
| M715T3 | 714.6 | 2.96 | DG(18:2(9Z,12Z)/22:5(4Z,7Z,10Z,13Z,16Z)/0:0) | HMDB07264 |
| M715T3 | 714.6 | 2.96 | DG(18:3(6Z,9Z,12Z)/22:4(7Z,10Z,13Z,16Z)/0:0) | HMDB07292 |
| M715T3 | 714.6 | 2.96 | DG(20:3(5Z,8Z,11Z)/20:4(5Z,8Z,11Z,14Z)/0:0) | HMDB07460 |
| M715T3 | 714.6 | 2.96 | DG(20:3(5Z,8Z,11Z)/20:4(8Z,11Z,14Z,17Z)/0:0) | HMDB07461 |
| M715T3 | 714.6 | 2.96 | DG(20:3(8Z,11Z,14Z)/20:4(8Z,11Z,14Z,17Z)/0:0) | HMDB07490 |

| | | | | |
|---|---|---|---|---|
| M715T3 | 714.6 | 2.96 | DG(20:4(5Z,8Z,11Z,14Z)/20:3(5Z,8Z,11Z)/0:0) | HMDB07516 |
| M715T3 | 714.6 | 2.96 | DG(20:4(5Z,8Z,11Z,14Z)/20:3(8Z,11Z,14Z)/0:0) | HMDB07517 |
| M715T3 | 714.6 | 2.96 | DG(20:4(8Z,11Z,14Z,17Z)/20:3(5Z,8Z,11Z)/0:0) | HMDB07545 |
| M715T3 | 714.6 | 2.96 | DG(20:4(8Z,11Z,14Z,17Z)/20:3(8Z,11Z,14Z)/0:0) | HMDB07546 |
| M715T3 | 714.6 | 2.96 | DG(20:5(5Z,8Z,11Z,14Z,17Z)/20:2(11Z,14Z)/0:0) | HMDB07573 |
| M715T3 | 714.6 | 2.96 | DG(22:4(7Z,10Z,13Z,16Z)/18:3(6Z,9Z,12Z)/0:0) | HMDB07684 |
| M715T3 | 714.6 | 2.96 | DG(22:4(7Z,10Z,13Z,16Z)/18:3(9Z,12Z,15Z)/0:0) | HMDB07685 |
| M715T3 | 714.6 | 2.96 | DG(22:5(4Z,7Z,10Z,13Z,16Z)/18:2(9Z,12Z)/0:0) | HMDB07712 |
| M715T3 | 714.6 | 2.96 | DG(22:5(7Z,10Z,13Z,16Z,19Z)/18:2(9Z,12Z)/0:0) | HMDB07741 |
| M715T3 | 714.6 | 2.96 | DG(22:6(4Z,7Z,10Z,13Z,16Z,19Z)/18:1(11Z)/0:0) | HMDB07768 |
| M715T3 | 714.6 | 2.96 | DG(22:6(4Z,7Z,10Z,13Z,16Z,19Z)/18:1(9Z)/0:0) | HMDB07769 |
| M715T3 | 714.6 | 2.96 | DG(18:0/20:4(8Z,11Z,14Z,17Z)/0:0) | HMDB07171 |
| M715T3 | 714.6 | 2.96 | DG(18:1(11Z)/20:3(8Z,11Z,14Z)/0:0) | HMDB07198 |
| M715T3 | 714.6 | 2.96 | DG(18:1(9Z)/20:3(5Z,8Z,11Z)/0:0) | HMDB07226 |

| | | | | |
|---|---|---|---|---|
| M715T3 | 714.6 | 2.96 | DG(18:3(6Z,9Z,12Z)/20:1(11Z)/0:0) | HMDB07282 |
| M715T3 | 714.6 | 2.96 | DG(18:4(6Z,9Z,12Z,15Z)/20:0/0:0) | HMDB07339 |
| M715T3 | 714.6 | 2.96 | DG(20:0/18:4(6Z,9Z,12Z,15Z)/0:0) | HMDB07367 |
| M715T3 | 714.6 | 2.96 | DG(20:1(11Z)/18:3(6Z,9Z,12Z)/0:0) | HMDB07394 |
| M715T3 | 714.6 | 2.96 | DG(20:1(11Z)/18:3(9Z,12Z,15Z)/0:0) | HMDB07395 |
| M715T3 | 714.6 | 2.96 | DG(20:2(11Z,14Z)/18:2(9Z,12Z)/0:0) | HMDB07422 |
| M715T3 | 714.6 | 2.96 | DG(20:3(5Z,8Z,11Z)/18:1(11Z)/0:0) | HMDB07449 |
| M715T3 | 714.6 | 2.96 | DG(20:3(5Z,8Z,11Z)/18:1(9Z)/0:0) | HMDB07450 |
| M715T3 | 714.6 | 2.96 | DG(20:3(8Z,11Z,14Z)/18:1(11Z)/0:0) | HMDB07478 |
| M715T3 | 714.6 | 2.96 | DG(20:3(8Z,11Z,14Z)/18:1(9Z)/0:0) | HMDB07479 |
| M715T3 | 714.6 | 2.96 | DG(20:4(5Z,8Z,11Z,14Z)/18:0/0:0) | HMDB07506 |
| M715T3 | 714.6 | 2.96 | DG(20:4(8Z,11Z,14Z,17Z)/18:0/0:0) | HMDB07535 |
| M715T3 | 714.6 | 2.96 | DG(22:4(7Z,10Z,13Z,16Z)/16:0/0:0) | HMDB07678 |
| M715T3 | 714.6 | 2.96 | DG(18:3(9Z,12Z,15Z)/20:1(11Z)/0:0)[iso2] | HMDB07311 |

| | | | | |
|---|---|---|---|---|
| M715T3 | 714.6 | 2.96 | PE(15:0/P-16:0) | HMDB08916 |
| M715T3 | 714.6 | 2.96 | PE(P-16:0/15:0) | HMDB11337 |
| M715T3 | 714.6 | 2.96 | lysoPC(28:1(5Z)) | HMDB29221 |
| M569T1 | 569.5 | 0.79 | Arachidonyl carnitine | HMDB06455 |
| M569T1 | 569.5 | 0.79 | LysoPC(20:1(11Z)) | HMDB10391 |
| M569T1 | 569.5 | 0.79 | LysoPC(20:0) | HMDB10390 |
