# Supplementary material for: Untargeted Plasma Metabolomics Reveals Metabolic Differences Associated with Job Stress in Manufacturing Workers: A Cross-Sectional Study
Source: Metabolites. 2026 Oct 18;16(10):790. doi: 10.3390/metabo16100790 (PMC13707163; doi:10.3390/metabo16100790)
Supplement: Supplementary file 1 [file metabolites-16-00790-s001.zip › metabolites-4479318-supplementary-Table S3.pdf]

Table S3. Apparent area under the receiver operating characteristic curves (AUCs) of the 12 differential features.

Metabolite	AUC	*P*	log2(FC)
M573T1	0.8522	<0.001	46.32
M264T3	0.7731	<0.001	6.85
M673T5	0.7641	<0.001	3.62
M686T27	0.7588	<0.001	24.68
M569T1	0.7125	0.002	16.74
M687T1	0.6958	<0.001	-11.79
M615T1	0.6868	<0.001	9.08
M589T2	0.6850	<0.001	10.50
M341T2	0.6794	<0.001	12.14
M659T1	0.6521	<0.001	7.75
M525T1	0.6509	<0.001	19.50
M715T3	0.5856	<0.001	7.99
