# Supplementary material for: Untargeted Plasma Metabolomics Reveals Metabolic Differences Associated with Job Stress in Manufacturing Workers: A Cross-Sectional Study
Source: Metabolites. 2026 Oct 18;16(10):790. doi: 10.3390/metabo16100790 (PMC13707163; doi:10.3390/metabo16100790)
Supplement: Supplementary file 1 [file metabolites-16-00790-s001.zip › metabolites-4479318-supplementary-Table S4.pdf]

Table S4. Metabolic pathway analysis

No	Pathway name	Total	Hits	*P*	FDR_q	Impact
1	Glycerophospholipid metabolism	36	3	<0.0001	0.008	0.22
2	Linoleic acid metabolism	5	1	0.0156	0.625	0.00
3	alpha-Linolenic acid metabolism	13	1	0.0402	1.000	0.00
4	Porphyrin metabolism	31	1	0.0938	1.000	0.03
5	Glycosylphosphatidylinositol (GPI）-anchor biosynthesis	32	1	0.0967	1.000	0.04
6	Steroid biosynthesis	41	1	0.1225	1.000	0.00
7	Arachidonic acid metabolism	44	1	0.1309	1.000	0.00
